# *Escherichia coli* avoids high dissolved oxygen stress by activation of SoxRS and manganese-superoxide dismutase

**DOI:** 10.1186/1475-2859-12-23

**Published:** 2013-03-12

**Authors:** Antonino Baez, Joseph Shiloach

**Affiliations:** 1 Biotechnology Core Laboratory, National Institute of Diabetes and Digestive and Kidney Diseases, National Institutes of Health, Bethesda, MD, 20892, USA

**Keywords:** Oxidative stress, ROS, SoxS, Catalase activity, SOD activity

## Abstract

**Background:**

High concentrations of reactive oxygen species (ROS) were reported to cause oxidative stress to *E*. *coli* cells associated with reduced or inhibited growth. The high ROS concentrations described in these reports were generated by exposing the bacteria to H_2_O_2_ and superoxide-generating chemicals which are non-physiological growth conditions. However, the effect of molecular oxygen on oxidative stress response has not been evaluated. Since the use of oxygen-enriched air is a common strategy to support high density growth of *E*. *coli*, it was important to investigate the effect of high dissolved oxygen concentrations on the physiology and growth of *E*. *coli* and the way it responds to oxidative stress.

**Results:**

To determine the effect of elevated oxygen concentrations on the growth characteristics, specific gene expression and enzyme activity in *E*. *coli*, the parental and SOD-deficient strain were evaluated when the dissolved oxygen (dO_2_) level was increased from 30% to 300%. No significant differences in the growth parameters were observed in the parental strain except for a temporary decrease of the respiration and acetate accumulation profile. By performing transcriptional analysis, it was determined that the parental strain responded to the oxidative stress by activating the SoxRS regulon. However, following the dO_2_ switch, the SOD-deficient strain activated both the SoxRS and OxyR regulons but it was unable to resume its initial growth rate.

**Conclusion:**

The transcriptional analysis and enzyme activity results indicated that when *E*. *coli* is exposed to dO_2_ shift, the superoxide stress regulator SoxRS is activated and causes the stimulation of the superoxide dismutase system. This enables the *E*. *coli* to protect itself from the poisoning effects of oxygen. The OxyR protecting system was not activated, indicating that H_2_O_2_ did not increase to stressing levels.

## Background

High density growth of *E*. *coli* is the preferred method for maximizing volumetric production yield of bacterial biomass and recombinant protein production [1,2]. Due to the high oxygen demand of such cultures, an efficient way to maintain aerobic growth conditions is to increase the oxygen concentration in the air supply by mixing the sparging air with pure oxygen [3-5]. In most cases, the oxygen concentration in the air inlet is increased from 20% to 68% [6] and in some cases as high as 100%. Previous research reported that oxidative stress created by exposing *E*. *coli* to H_2_O_2_ and superoxide-generating chemicals was found to be detrimental to the bacterial growth [7-12]. However, the effects of high concentration of molecular oxygen on the bacterial physiological and molecular response either in shake flask growth or at high density growth conditions in bioreactors are unknown.

During its biological reduction to water through respiration, molecular oxygen is generating reactive oxygen species (ROS) such as superoxide anion (O_2_^‒^), hydrogen peroxide (H_2_O_2_), and hydroxyl radicals (HO*) [13-15]. High levels of ROS are known to be stress conditions for *E*. *coli* causing irreversible damage to cellular components [15,16]. To prevent this, *E*. *coli* is equipped with a defense mechanism regulated by the SoxRS and OxyR regulons [7,15,17,18]. At normal growth conditions, SoxR is produced in an inactivated (reduced) form, but when exposed to superoxide or redox-cycling drugs, SoxR is activated together with simultaneous activation of the the *soxS* gene [8,9,19]. The SoxS protein is a secondary transcription factor that activates the expression of the following genes: *sodA*, *acnA*, *fumC*, *micF*, and *zwf*, replacing sensitive enzymes such as aconitase B and fumarases A and B with the oxygen resistant isozymes aconitase A and fumarase C [20-23]. The OxyR regulon is mainly activated by H_2_O_2_, enhancing the transcription of a set of genes that increase hydrogen peroxide resistance. These include the *katG*, *ahpCF*, *gor*, *grxA*, *trxC* and *OxyS* genes [7,10,15].

The purpose of this work was to evaluate the effect of elevated oxygen concentration on the growth characteristics, enzyme activities, and expression of genes related to the SoxRS and OxyR regulons in *E*. *coli* growing in bioreactors. The findings explain the bacterial defense mechanism to high molecular oxygen concentrations in bioreactors.

## Results

### Effect of 300% oxygen saturation on growth, respiration and acetate production of *E*. *coli* MG1655

**Figure 1:**
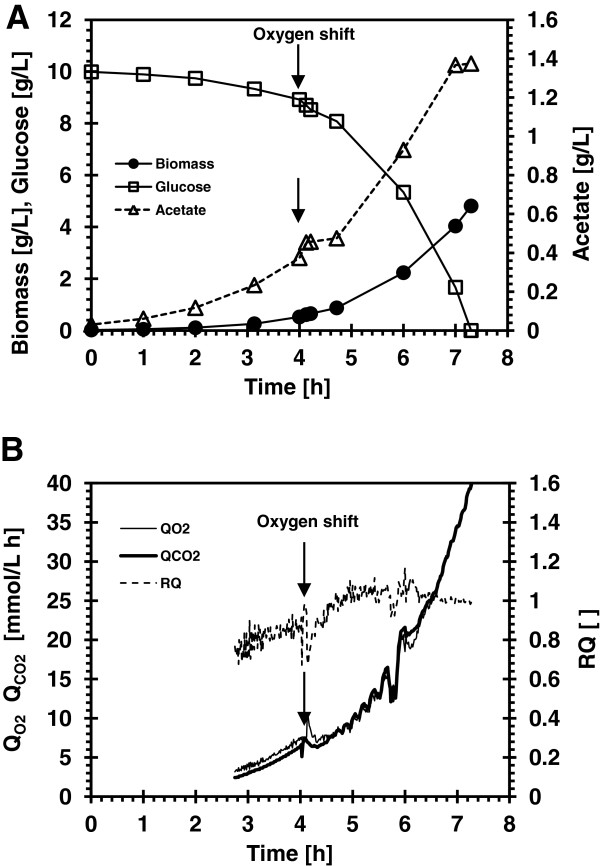
**Batch growth of *E. coli *MG1655 at 30% and 300% dO**_**2**_**.** (**A**) Time course of biomass, glucose and acetate concentrations. (**B**) Time course of the volumetric oxygen uptake and carbon dioxide formation rates and the respiratory quotient. The arrow indicates when dO_2_ was increased from 30% to 300%.

**Figure 2:**
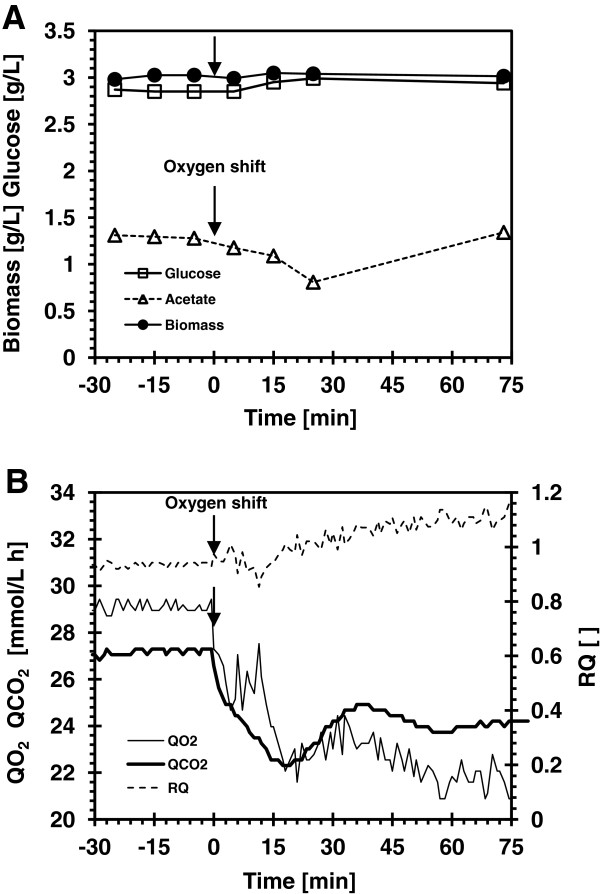
**Glucose-limited continuous culture of *E. coli *MG1655 at 30% and 300% dO**_**2**_**.** (**A**) Biomass concentration, residual glucose and excreted acetate concentration (**B**) Volumetric oxygen uptake rate (Q_O2_), carbon dioxide formation (Q_CO2_) rate, and the respiratory quotient (RQ) profile. The arrow indicates when dO_2_ was increased from 30% to 300%.

The effect of dissolved oxygen (dO_2_) concentration of 300% air saturation on the growth, respiration, glucose consumption and acetate production of *E*. *coli* K strain (MG1655) was studied in batch and chemostat cultures. The results of the batch growth are summarized in Figure 1 and Table 1 and those of the chemostat cultures in Figure 2 and Table 2. In the batch growth, the cells grew initially at dO_2_ concentration of 30% air saturation; when the culture density reached an OD_600_ of 1, the dO_2_ was increased to 300% and the culture was kept at that level throughout the growth. Following the change in the dO_2_ concentration, there was a short perturbation in the acetate production profile (Figure 1A), slight decrease in CO_2_ production and oxygen consumption rates, and an increase in the respiratory quotient (RQ) (Figure 1B). However, no significant changes (p = 0.05) were observed in growth characteristic and overall yield coefficients of the culture (Table 1). To quantify the magnitude of the dO_2_ effect on the respiration and acetate profile observed in batch cultures, a glucose-limited chemostate culture of *E*. *coli* MG1655 operated at steady state (D = 0.5 h^-1^) was performed. Thirty minutes after the change in the dO_2_ concentration, acetate concentration decreased from 1.3 to 0.8 g/L, and it came back to its initial value within an hour; the biomass and the glucose concentrations were unchanged (Figure 2A). Following the dO_2_ change the oxygen uptake rate decreased by 25% and carbon dioxide evolution rates decreased by 11%; as was observed in the batch cultures the RQ in the chemostat increased by 19% (Figure 2B). Table 2 summarizes the growth characteristic of the chemostat culture following the change in dO_2_ concentration. The increase in RQ suggests that more glucose was directed to the assimilatory pathway for biomass production and correlates with the slight increase in the glucose utilization yield Y_x/s_. The respiration changes observed in the chemostat culture did not affect the growth characteristics of *E*. *coli* MG1655, similar to what was observed in the batch culture. Supplying pure oxygen to the chemostat culture did not change further the growth rate but decreased acetate accumulation by 16% and glucose consumption by 10%, (results not shown). The sudden increase in the dissolved oxygen concentration had a minimal effect on *E*. *coli* MG1655 growth.

**Table 1:** **Batch growth parameter of *E. coli *MG1655 at dO2 of 30% and 300%**

	**Culture exposed to 300% dO**_**2**_	**Reference culture (30% dO**_**2**_**)**	**% Change**
Specific growth rate, μ,(h-1)	0.684 ± 0.105	0.717 ± 0.038	−4.6
Glucose uptake rate, q_s_, (g/g.h)	1.34 ± 0.213	1.45 ± 0.059	−7.6
Biomass yield, Y_x/s_, (g/g)	0.515 ± 0.082	0.496 ± 0.037	3.8
Acetate yield, Y_A/s_, (g/g)	0.139 ± 0.005	0.136 ± 0.010	2.2
Maximum acetate conc. (g/L)	1.41 ± 0.098	1.40 ± 0.100	0.7

**Table 2:** **Continuous culture growth parameters of *E. coli *MG1655 before and after oxygen switch from 30%dO2 to 300% dO2**

	**Prior to O**_**2 **_**switch**	**After O**_**2 **_**switch**	**% Change**
qO_2_, (mmol/g h)	9.65	7.25	−24.9
qCO_2_, (mmol/g.h)	9.05	8.03	−11.3
RQ	0.94	1.12	19.1
q_acetate_, (mmol/g h)	3.58	2.82	−21.2
q_s_, (mmol/g h)	5.10	4.96	−2.7
Y_x/s_, (g/g)	0.54	0.56	3.7

### Effect of 300% oxygen saturation on superoxide dismutase and catalase activity in *E*. *coli* MG1655

**Figure 3:**
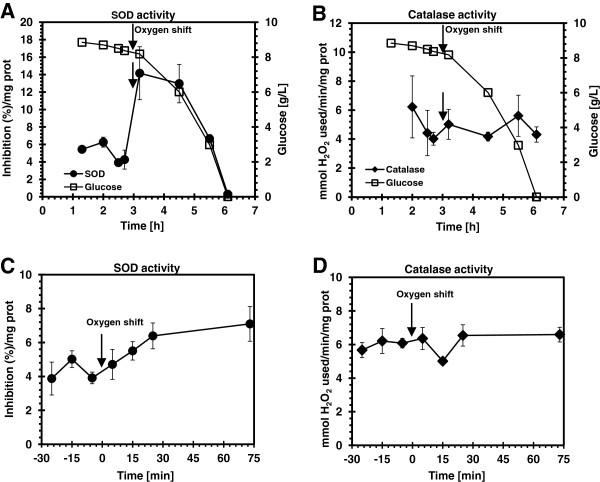
**Superoxide dismutase (SOD) and catalase activity in *E. coli *MG1655 at 30% and 300% dO**_**2**_**.** (**A**) SOD and (**B**) Catalase activity in a batch culture (**C**) SOD and (**D**) Catalase activity in a glucose-limited continuous culture (D = 0.5 h^-1^). The arrows indicate when dO_2_ was increased from 30% to 300%. Error bars represent standard deviations between triplicate analyses of the same sample.

The exposure of *E*. *coli* MG1655 to 300% dO_2_ concentration triggered short perturbation period of decreased respiration, acetate accumulation, and glucose consumption, suggesting limited stress conditions. To investigate further how the bacteria respond to the abrupt dissolved oxygen change, the enzymatic activities of superoxide dismutase (SOD) and catalase were measured. The results for the batch and chemostat cultures are shown in Figure 3. The SOD activity in the batch culture increased 2.3 fold in response to the oxygen shift and decreased towards the end of the growth (Figure 3A). At the same time catalase activity was not affected by the increase in dO_2_ and was the same throughout the entire cultivation period (Figure 3B). To exclude the effect of varying media composition on SOD and catalase activities, a chemostate culture was performed and the enzymatic activities of SOD and catalase were measured before and after the dO_2_ shift from 30 to 300% (Figure 3C, D). The enzymatic activities were similar to those observed in the batch culture; SOD activity was stimulated by the increase in dO_2_ concentration but catalase activity stayed the same.

### Effect of high oxygen saturation on gene transcription in the SoxRS and OxyR regulons in *E*. *coli* MG1655

**Figure 4:**
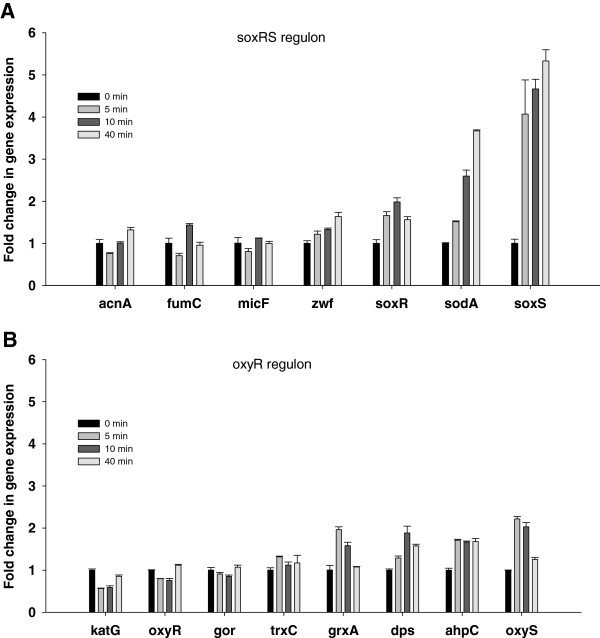
**Time course expression of selected genes controlled by SoxRS and OxyR regulons during *E. coli *growth at 30% and 300% dO**_**2**_**.** (**A**) SoxRS controlled genes, (**B**) OxyR controlled genes, changes in mRNA were analyzed 0, 5, 10, and 40 min after dO_2_ shift from 30% to 300% in *E*. *coli* MG1655. Error bars represent standard deviations between triplicate analyses.

Time-course transcription analysis of selected genes associated with SoxRS and OxyR regulons in *E*. *coli* MG1655 was conducted following the increase in dO_2_ concentration; the results are summarized in Figure 4. Forty minutes after the dO_2_ shift, the transcription of the *soxS* regulator was 5.3 fold higher, of *sodA* (Manganese superoxide dismutase) 3.7 fold higher, of *soxR* (Superoxide response protein) and of *zwf* (Glucose 6 phosphate dehydrogenase) 2 fold higher The oxygen shift did not increase the expression of the oxidant-resistant isozymes aconitase A and fumarase C which are encoded by the *acnA* and *fumC* genes and the small regulatory RNA micF (Figure 4A). The transcription of genes under the control of OxyR regulon was notably less affected than those of the SoxRS regulon (Figure 4B). Transcription levels of *grxA*, *dps*, *ahpC*, and *oxyS* (whose products are glutaredoxin I, stress response DNA binding protein, alkyl hydroperoxide reductase, and regulatory RNA respectively), were increased no higher than 2-fold.

### Effect of high oxygen saturation on specific growth rate and gene transcription in the *E*. *coli* AB1157 and the double mutant strain *sodA*^―^*sodB*^―^

**Figure 5:**
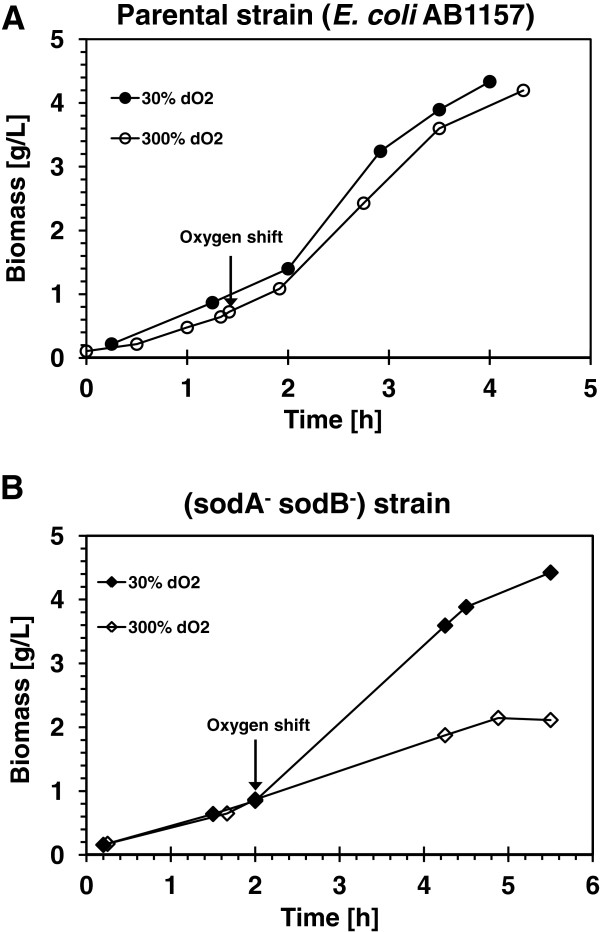
**Effects of dissolved oxygen shift on growth of *E. coli *AB1157 and SOD deficient mutant.** The arrows indicate when dO_2_ was increased from 30% to 300%. The reference culture was labeled as 30% dO_2_ (solid symbols).

The effect of deleting the *sod* gene on the *E*. *coli* ability to respond to increase in oxygen concentration from 30% to 300% was evaluated by following the growth patterns of the parental *E*. *coli* AB1157 strain and the double mutant strain PN134 (*sodA*^‒^*sodB*^‒^). The growth patterns are shown in Figure 5; Figure 5A shows no effects of oxygen shift on the growth behavior of parental strain. The specific growth rates were 1.08 h^-1^ for the control growth and 1.06 h^-1^ for the culture expose to the increase in the dO_2_. On the other hand The SOD deficient strain was affected; following the dO_2_ shift its specific growth rate was reduced from 0.75 h^-1^ at 30% to 0.32 h^-1^ at high dO_2_ (Figure 5B).

**Figure 6:**
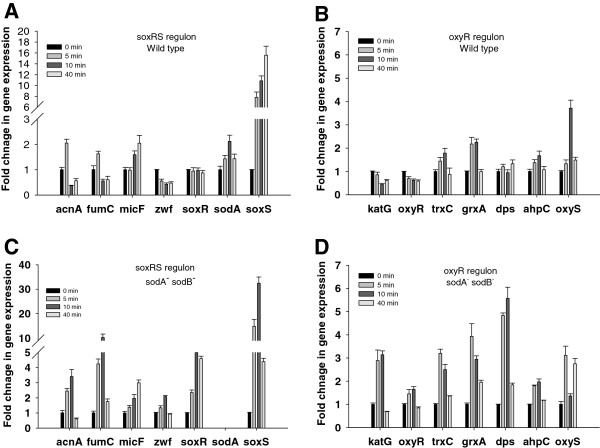
**Time point expression of selected genes controlled by SoxRS and OxyR regulons in *E. coli *AB1157 and SOD deficient mutant.** (**A**), (**C**) SoxRS-controlled genes, (**B**), (**D**) OxyR-controlled genes. Relative changes in mRNA were evaluated 0, 5, 10, and 40 min after dO_2_ increased from 30% to 300%. Error bars represent standard deviations of three repetions.

Transcriptional analyses of the SoxRS regulon shows that the dO_2_ shift in the *E*. *coli* AB1157 increased the transcription of *soxS* by 16 fold, the transcription of *sodA* by 2.1 fold, but did not have any effect on the rest of the SoxRS-controlled genes (Figure 6A). The transcription analysis of the OxyR regulon shows that following the exposure to high dO_2_, the transcription of both *trxC* and *grxA* increased by 2.2 fold and the transcription of *oxyS* by 3.7 fold (Figure 6B). Different patterns of gene expression were observed in the double mutant strain *sodA*^‒^*sodB*^‒^. In the SoxRS regulon, the genes that encode for the oxidant-resistant isozymes, *acnA* and *fumC*, were up-regulated 3.4- and 10.2-fold respectively, suggesting that aconitase B and fumarases A and B were inactivated by the oxidative stress. The expression of *soxS* in the double mutant strain increased by 32.5 fold following the change in dO_2_ which was significantly higher than the response observed in the *E*. *coli* AB1157 strain (Figure 6A, C). Concerning the transcription of the OxyR regulon, both *katG* and *trxC* genes were up-regulated by 3.2 fold, whereas *grxA* and *dps* were up-regulated by 4 and 5.6 fold respectively (Figure 6D). Compared with the parental strain, the double mutant showed higher activity of SoxRS and OxyR transcriptional factors suggesting stronger oxidative stress response in this strain. To test if that could be due to a higher ROS accumulation in the double mutant, intracellular ROS concentrations were measured in parental and SOD deficient strains (Table 3). Although the ROS concentration was higher at 300% dO_2_ than at 30% of dO_2_ there was no difference between the parental and the SOD deficient strain.

**Table 3:** **Effects of dissolved oxygen on intracellular ROS concentration in *E*. *coli***

	**DFC production relative to 30% dO**_**2 **_**condition**	
	**Parental (AB1157)**	**Deficient strain (sodA- sodB-)**
N_2_	-	0.45 ± 0.042
30% dO_2_	1.00 ± 0.029	1.00 ± 0.018
300% dO_2_	1.22 ± 0.041	1.22 ± 0.048
2 mM H_2_O_2_	2.71 ± 0.138	2.59 ± 0.050

The data shown are the means and standard deviation of three determinations.

## Discussion

It has been reported that exposing *E*. *coli* to reactive oxygen species (ROS), generated by chemical compounds such as H_2_O_2_ and paraquat, causes damage to the growing cells by reducing DNA stability and modifying proteins and lipids [15,16,24]. Since efficient growth of *E*. *coli* for production of recombinant proteins is based on growing the bacteria to high density by supplying air mixed with pure oxygen [3-5], it was essential to investigate the effect of high dissolved oxygen concentrations on bacterial growth.

The dissolved oxygen concentration is usually kept at 30% dO_2_. In this reported work, the effect of higher dissolved oxygen concentrations on the bacterial growth and gene expression was evaluated. The data shows that 300% dO_2_ did not affect the growth rate of the bacteria as a result of the activation of the manganese-superoxide dismutase (Mn-SOD), which is part of the activated SoxRS regulon. But high dO_2_ had small and short time effect on the respiration, the acetate accumulation, SOD activity and *soxS* expression suggesting sub-lethal damage to the *E*. *coli* culture.

A possible trigger for the activation of *soxS* expression is the high intracellular levels of O_2_ that stimulate SoxR oxidation by abstracting electrons from flavoenzymes and promoting the increased level of reactive oxygen species [15]. The increased ROS concentration decreased the NADPH/NADP^+^ ratio, which was suggested to be the signal for SoxRS activation [19,25,26]. It is also possible that hyperbaric oxygen (4.2 atm of pO_2_) blocks the synthesis and decreases intracellular concentrations of NAD and NADP [21,27,28].

Hyperbaric oxygen destroys the [4Fe-4S] cluster in dihydroxyacid dehydratase (DHAD), and inactivates the enzyme [29]. Inactivated DHAD limits the biosynthesis of branched-chain amino acids and triggers stringent response [30,31]. In minimal medium without supplementation of amino acids, the inactivation of DHAD causes growth inhibition [31]. It is therefore likely that the oxidative stress generated in our study did not affect the DHAD activity since there was no significant change in the bacterial growth even when defined medium without supplementation of branched-chain amino acids was used. Hyperbaric oxygen also inactivates fumarase A and B, both containing the catalytically active 4Fe-4S cluster [29], but in our study the activity of these oxidant-sensitive enzymes was not impaired by the dO_2_ shift since the oxidant-resistant isozyme fumarase C was not activated. These results could be explained by the protecting effects of superoxide dismutases on DHAD and fumarases [29,30] and is in agreement with the up-regulation of *sodA* and increased SOD activity. The same protecting effect of Mn-SOD on aconitase stability has been observed during exposure to hyperoxia [21] which could explain why the expression of the superoxide-resistant aconitase A was not required.

Unlike the parental strain AB1157, the *sodA*^‒^*sodB*^‒^ strain requires the expression of the oxidant-resistant dehydratases, (aconitase A and fumarase C) probably as the result of the inactivation of aconitase B and fumarase A and B by the presence of O_2_^‒^[29,30]. In our study the possible increased level of O_2_^‒^ inside the deficient strain *sodA*^‒^*sodB*^‒^ activated also the OxyR-controlled genes, likely due to the production of extremely potent oxidants derived from ferric complexes and nitric oxide as a result of the missing SOD [32]. This assumption is supported by the fact that superoxide can reduce a variety of ferric complexes and increase the availability of Fe^+2^ which catalyzes Fenton and Haber-Weiss reactions [16,32]. The parental and the double mutant *sodA*^‒^*sodB*^‒^ strains did not show any difference in intracellular ROS accumulation. This is likely the result of the inability of the fluorimetric assay to detect differences in ROS concentrations in the range of 0.1 nM [33] which is calculated to be the physiological concentration of superoxide [34]. However, the damage caused by this small change of superoxide in the SOD-deficient strain significantly affects its genes expression and growth.

Based on the presented work, we determined that *E*. *coli* can successfully prevent the toxic effects of high oxygen saturation through the activation of SoxRS and the over-expression of manganese-superoxide dismutase in response to increased concentration of intracellular ROS. The use of molecular oxygen in growing *E*. *coli* does not affect growth properties but because of the possible sub-lethal effects, its potential effects on quality of recombinant protein production and culture stability should be considered.

## Conclusions

Increasing dissolved oxygen concentration during *E*. *coli* growth stimulates an increase in intracellular ROS concentration that activates the SoxRS regulon. The activation of the regulon is associated with over expression of manganese-superoxide dismutase that scavenges the O_2_^-^ and prevents irreversible damage to the growing cells. SoxRS and SOD are identified as the main defense mechanism that protects the bacteria from the toxic effects of high oxygen saturation.

## Methods

### Bacterial strains, inoculum preparation, and culture media

*Escherichia coli* MG1655 (F-, λ, ilvG-, rfb-50, rph-1) was grown in batch and continuous bioreactor cultivations in the following medium: KH_2_PO_4_, 12.5 g/L; (NH_4_)_2_HPO_4_, 5 g/L; citric acid, 1.0 g/L. The pH of the medium was adjusted to 7.0 with 5 M NaOH prior to sterilization, after sterilization the media was aseptically supplemented with 1 mL/L trace metal solution [3], 5 mM MgSO_4_, 4.5 mg/L thiamine-HCl, and 8 g/L glucose. *E*. *coli* strains AB1157 (Parental), PN134 (*sodA sodB* deficient) provide by Dr. James A. Imlay [8] were grown in modified LB medium containing 10 g/L tryptone, 5 g/L yeast extract, 5 g/L NaCl, and 5 g/L K_2_HPO_4_, and supplemented with 1 mL/L of trace metal solution, 5 mM MgSO_4_, 4 g/L of glucose and appropriate antibiotic after sterilization. Inoculums were prepared by growing the cultures at 37°C in 100 mL of defined or complex medium.

### Bacterial growth

Batch growth was performed in a 5 L B. Braun bioreactor equipped with data acquisition and adaptive dissolved oxygen control system. Temperature was maintained at 37°C and was maintained at pH 7.0 by the addition of 3 M NaOH. Bioreactor was inoculated at OD_600_ of 0.1 and the dissolved oxygen (dO_2_) was measured with polarographic oxygen electrode (Mettler Toledo, Columbus, OH) and maintained at 30% air saturation. When the cell growth reached an OD_600_ of 1 the dO_2_ was increased to 300% air saturation by mixing the air supply with pure oxygen and was maintained at this value by increasing agitation and gas flow rate. A dO_2_ of 300% air was chosen since it is equivalent to 63% of O_2_ in the inlet gas. Such oxygen concentration in the gas supply can be easily reached in a typical bench scale fed-batch culture [6].

The dissolved oxygen sensor used (InPro 6800 Mettler Toledo) was calibrated with nitrogen at zero and with pure O_2_ at 100%. After calibration and before inoculation, air was bubbled into the medium to verify that the dO_2_ was 21%. To maintain the culture at 30% air saturation, a set-point of 6% was set up and oxygen shift was made by changing the set-point to 63%. The concentrations of O_2_ and CO_2_ in the inlet and outlet of the bioreactor were analyzed by mass spectrometry gas analyzer (Perkin Elmer MGA 1200). The analyzer performance was verified by pumping gas mixtures containing 21%, 63% and 100% O_2_, the values measured by gas analyzer were 21.4%, 63.6% and 98.5% respectively. In addition the CO2 measurement was verified with 5% CO_2_. During the experiments at 300% DOT, oxygen concentration in the off gas varied from 62 to 64% and CO_2_ concentration did not exceed 3.4%. Samples for RNA analysis were collected at 0, 5, 10, and 40 minutes after the increase of the dO_2_ and processed immediately, media samples were collected at regular intervals and centrifuged at 10000 × g for 5 min and the supernatant was kept at -20°C for further analysis. The cell pellet was washed twice in 50 mM sodium phosphate buffer pH 7.4 and kept at -20°C for enzyme assays. The continuous culture was carried out at a dilution rate of 0.5 h^-1^ at the same growth conditions. After five residence times when steady state was confirmed the culture was perturbed by increasing the dO_2_ from 30% to 300% air saturation, samples for enzyme assays were taken 5, 15, 25 and 74 min after oxygen shift.

### Analytical methods

Cell growth was followed by measuring the OD at 600 nm (Ultrospec 3000 UV/Visible spectrophotometer, Pharmacia Biotec); measurements were converted to dry cell weight by using a calibration curve of dried samples. Glucose concentration was determined by YSI 2700 Biochemistry Analyzer (YSI Instruments, Yellow Springs, OH). Organic acids were analyzed by high-performance liquid chromatography (Hewlett Packard/Agilent 1100 Series, Santa Clara, CA) with an Aminex HPX-87H column (Bio-Rad Laboratories, Hercules, CA) at 35°C using mobile phase of 5 mM H_2_SO_4_ at 0.6 ml/min.

### RT-qPCR analysis

For real-time RT-qPCR determinations, fermentation samples were immediately poured on an ice-cooled tube containing RNAlater solution (Ambion Inc. Austin, TX). Total RNA extraction was performed using mirVana miRNA isolation kit (Ambion Inc. Austin, TX). Total RNA concentration was estimated by measuring optical density at 260 nm using NanoDrop 2000/2000c spectrophotometer (Thermo Fisher Scientific, Wilmington, DE) and integrity was visualized on a 2% agarose gels. To reduce genomic DNA contamination, isolated RNA was treated with turbo DNase kit (Ambion Inc. Austin, TX). cDNA was generated by using 2 μg RNA in a total volume of 20 μl with 250 nM of specific DNA primers (antisense primers in Additional file 1: Table S1) according to the protocol of Maxima First Strand cDNA Synthesis kit (Thermo Fisher Scientific, Waltham, MA). Real-time qPCR was performed using an ABI Prism 7900H Sequence Detection System (Applied Biosystems, Foster City, CA) with 40 amplification cycles using SYBR Green PCR Master Mix as signal reporter. Each reaction composed of 6 ng cDNA, 400 nM sense and antisense primers in a total volume of 20 μl. RT-qPCR was done in a 96-well microtiter PCR plates using the following amplification conditions: 1 cycle 10 min at 95°C; and 40 two-step cycle at 95°C for 15 seconds and 60°C for 60 seconds. Each sample was done in triplicate. To assess for reagent and genomic DNA contamination, no template and no reverse transcriptase controls were included. Data were analyzed using 2^-ΔΔCT^ method described by Livak and Schmittgen [35]. The expression of the *ssrA* gene was used as an endogenous control to normalize the amount of mRNA obtained from a target gene [36]. Expression data obtained for each time-point were normalized to the expression of each gene obtained at time zero of the oxygen switch.

### Cell-free extracts and enzymes assays

Frozen cells were suspended in 300 μl of 50 mM sodium phosphate buffer (pH 7.4 at 4°C) and disrupted by sonication in series 4 × 15 seconds in a cold bath. The extract was centrifuged at 10,000 × g at 4°C for 15 min and the supernatant was immediately used for enzyme assays. Protein concentrations were determined by using the method of Bradford. Catalase activity was assayed spectrophotometrically by measuring the decrease in A_240_ nm of 4 mM H_2_O_2_ in 50 mM phosphate buffer pH 7.4 [37]. Superoxide dismutase activity was determined using the Sigma SOD assay kit-WST (Sigma-Aldrich, Switzerland) following the manufacturer’s instructions.

### Measurement of intracellular level of ROS

OxiSelect Intracellular ROS assay kit (Cell Biolabs, Inc. San Diego, CA) was used according to the manufacturer’s instructions with minor modifications. AB1157 and PN134 strains growing in exponential phase were washed and incubated with 2’, 7’-dichlorodihydrofluorescin diacetate for 60 min at 37°C and washed three times with PBS buffer. The 2’,7’-dichlorofluorescein diacetate-loaded cells were re-suspended in 2 ml LB media and then exposed to 2 mM of H_2_O_2_ or bubbling with a gas mix corresponding to 0%, 30%, and 300% dO_2_ air saturation for 20 min. The treated cells were lysed and the amount of intracellular ROS was estimated from dichlorodihydrofluorescein (DCF) production measured at 480-nm excitation/530-nm emission by a SpectraMax Gemini XS Fluorometer plate reader (Molecular Devices, Sunnyvale, CA).

## Competing interests

The authors declare that they have no competing interests.

## Authors’ contributions

AB designed and conducted the experiments. AB and JS together analyzed the data and wrote the manuscript. Both authors read and approved the final manuscript.

## Supplementary Material

Additional file 1: Table S1 Set of primers used for quantitative real-time PCR amplification assays. Click here for file
