# Supplementary material for: Escherichia coli avoids high dissolved oxygen stress by activation of SoxRS and manganese-superoxide dismutase
Source: Microb Cell Fact. 2013 Mar 12;12:23. doi: 10.1186/1475-2859-12-23 (PMC3605374; doi:10.1186/1475-2859-12-23)
Supplement: Additional file 1: Table S1 — Set of primers used for quantitative real-time PCR amplification assays. [file 1475-2859-12-23-S1.docx]

**Additional files**

**Additional file 1 Table S1 Set of primers used for reverse transcriptase-PCR (antisense primers) and quantitative real-time PCR amplification (sense and antisense primers) assays.**

| **Gene name** | **Gene product** | **Sense primer/(Antisense primer)** |
| --- | --- | --- |
| **acnA** | Aconitase A | 5-AGGACTTTACCGGCGTACCT-3/(5-CATTTCCAGGCGTACGTTTT-3) |
| **ahpC** | Alkyl hydroperoxide reductase | 5-AAACCAGGCATTCAAAAACG-3/(5-TGCTTTGTGGGTGAAGTGAG-3) |
| **dps** | Nonspecific DNA binding protein | 5-ATGAAATGCTGGATGGCTTC-3/(5-GGTCAGCCAGTTCTTTCAGG-3) |
| **fumC** | Fumarase C | 5-CATTACCTGTGCACCGTTTG-3/(5-CTCATTTTCCGGGATTGAGA-3) |
| **gor** | Glutathione reductase | 5-GCCGATTGGTACTGTTGGTT-3/(5-AAAGCCAATGCCGTGAATAC-3) |
| **grxA** | Glutaredoxin 1 | 5-CCGTTATTTTTGGTCGTTCG-3)/(5-GCACGGTTTCTACGGGTTTA-3) |
| **katG** | Hydroperoxidase I | 5-CTGGTGTGGTTGGTGTTGAG-3/(5-AGTGACTCGGTGGTGGAAAC-3) |
| **micF** | RNA regulator of ompF | 5-GCTATCATCATTAACTTTATTTATTACCGT-3/(5-GGGTAAACAGACATTCAGAAGTGA-3) |
| **oxyR** | Oxidative stress regulator | 5-CATTCATTGAAGTGCCGTTG-3/(5-CGCGGAAGTGTGTATCTTCA-3) |
| **oxyS** | Regulatory RNA | 5-GGAGCGGCACCTCTTTTAAC-3/(5-ATCCTGGAGATCCGCAAAAG-3) |
| **sodA** | Manganese superoxide dismutase | 5-GCCTGTTCTGGAAAGGTCTG-3/(5-CCAGTTTATCGCCTTTCAGC-3) |
| **soxR** | Superoxide response protein | 5-GTATCCGTAACAGCGGCAAT-3/(5-CATTGGGACGAAAGCTGTTT-3) |
| **soxS** | Regulation of superoxide response regulon | 5-TTATCGCATGGATTGACGAG-3/(5-ACATAACCCAGGTCCATTGC-3) |
| **ssrA** | Transfer-messenger RNA (endogenous control) | 5-TTAGGACGGGGATCAAGAGA-3/(5-GCGTCCGAAATTCCTACATC-3) |
| **trxC** | Thioredoxin 2 | 5-CACGACTTGTTTGACGGAGA-3/(5-TCAATTCACGTTCAGCTTCG-3) |
| **zwf** | Glucose-6-phosphate dehydrogenase | 5-TGGCCTTGACCACAAACATA-3/(5-CAGGCTTCTTCCACTTCGTC-3) |
